# Self-Supported Defect-Rich Au-Based Nanostructures as Robust Bifunctional Catalysts for the Methanol Oxidation Reaction and Oxygen Reduction Reaction in an Alkaline Medium

**DOI:** 10.3390/nano11092193

**Published:** 2021-08-26

**Authors:** Yuanyuan Tao, Xiu Liang, Guanchen Xu, Dongwei Li, Yong Li, Na Zhang, Yingzhou Chen, Xifeng Jiang, Hongyu Gong

**Affiliations:** 1 Advanced Materials Institute, Qilu University of Technology (Shandong Academy of Sciences), Jinan 250014, China; yytao@sdas.org (Y.T.); xliang@sdas.org (X.L.); gcxu@sdas.org (G.X.); dwli@sdas.org (D.L.); yongli@sdas.org (Y.L.); 2 School of Chemistry and Chemical Engineering, Shandong University, Jinan 250100, China; 3 Shandong Gold Smelting Co., Ltd., Laizhou 261441, China; chenyingzhou1989@163.com; 4 Gweike Tech Co., Ltd., Jinan 250100, China; koki0201@163.com

**Keywords:** AuCu alloy, surface defects, electrochemical Cu removal, methanol oxidation reaction, oxygen reduction reaction

## Abstract

Recently, alkaline direct methanol fuel cells have made great progress with the development of alkaline electrocatalysis, and a wide variety of catalysts have been explored for methanol oxidation reaction (MOR)and oxygen reduction reaction (ORR). However, the slow kinetics of the MOR and ORR remain a great challenge. In this paper, self-supported defect-rich AuCu was obtained by a convenient one-pot strategy. Self-supported AuCu presented a branched, porous nanostructure. The nanobranch consisted of several 13 nm skeletons, which connected in the kink of the structure. Different growth directions co-existed at the kink, and the twin boundaries and dislocations as defects were observed. When the Au-based nanostructure functioned as an electrocatalyst, it showed robust MOR and ORR performance. For the MOR, the forward peak current was 2.68 times greater than that of Au/C; for the ORR, the activity was close to that of Pt/C and significantly better than that of Au/C. In addition, it possessed superior electrochemical stability for MOR and ORR. Finally, an in-depth exploration of the impact of surface defects and electrochemical Cu removal on MOR and ORR activity was carried out to explain the MOR and ORR’s catalytic performance.

## 1. Introduction

Many problems encountered in hydrogen fuel cell technology, particularly those of hydrogen storage and distribution, can be circumvented by replacing hydrogen with liquid fuels, such as methanol [1]. Two important reactions in direct methanol fuel cells, the methanol oxidation reaction (MOR) and the oxygen reduction reaction (ORR), have received great attention in recent years [2]. Along with the clean energy conversion, the slow kinetics of MOR and ORR seriously restrict the progress of electrocatalysis; hence, efficient catalysts are necessary for MOR and ORR [3]. In an acidic medium, for MOR, Pt/Rh are representative highly active catalysts [4,5,6,7]. Pt, as the most common and efficient catalyst, has been widely investigated [4,5], and hydroxide species forming on the surface Rh sites facilitate CO oxidation [6,7]. For ORR, Pt/Pd catalysts are known to exhibit excellent activity due to their appropriate oxygen and hydroxyl adsorption energies [8,9]. Recently, alkaline direct methanol fuel cells have made great progress with the development of alkaline electrocatalysis, and a wide variety of catalysts have been explored for MOR and ORR [10,11]. However, for most alternatives, they are only active enough for one of the two reactions. Carbon-based materials, for example, exhibit high ORR activity, while their MOR activity is quite limited [12,13]. Hence, the research and development of bifunctional catalysts in an alkaline medium for MOR and ORR is of great importance. As a potential candidate, Au-based catalysts have the advantages of more natural reserves and higher conductivity than Pt, but the intrinsic activity is very poor, which needs to be strengthened by a variety of methods [14,15,16,17]. For example, one important strategy is the fabrication of gold structures with abundant defects and highly active low-coordinated atoms, such as steps and kinks, which could serve as excellent catalytic sites for MOR and ORR [14,16]. In addition, the morphology and electronic structure of Au-based catalysts can be further controlled by introducing a second and third transition metal to synthesize Au-based alloys, thus enhancing the electrocatalytic MOR and ORR activity of Au [15,17].

Meanwhile, the removal of transition metals on the surface of Au-based alloys during electrocatalysis is also important. In some studies, the electronic structure of Au can be further optimized through the dealloying process [18]. In other cases, the removal of transition metals at the surface will affect the adsorption and desorption properties of intermediate species [19]. Therefore, through the research of surface transition metals’ removal, the catalytic mechanism of Au-based alloy catalysts can be further understood. Here, we report a facile method to synthesize self-supported AuCu. The product showed a highly efficient MOR and ORR catalytic performance, and the catalytic mechanism of MOR and ORR was studied by analyzing the state of the catalyst surface.

## 2. Materials and Methods

All reagents were used without further purification. The reagents’ characterization and electrochemical measurements can be found in the Supporting Information.

### 2.1. Synthesis of AuCu

In a typical synthesis, octadecylamine (ODA, 1.9 g), a HAuCl_4_∙4H_2_O solution (8.32 mL, 24.3 mM), and CuCl_2_∙2H_2_O (0.0341 g) were dissolved in a mixed solvent containing 20 mL dimethyl sulfoxide (DMSO) and 42.48 mL H_2_O. The mixture was magnetically stirred at 25 °C for 6 h. The resulting suspension was transferred to an autoclave and thermally treated at 120 °C in an electric oven for 48 h. After the thermal treatment, the resulting sample was separated by centrifuging, repeatedly washed with water and ethanol, and finally dried naturally overnight.

### 2.2. Synthesis of Au/C

Au nanoparticles of about a 13 nm diameter loaded on Ketjen black were prepared by the reduction of HAuCl_4_ with trisodium citrate [20,21]. In a typical procedure, 4 mL of 24.3 mM HAuCl_4_ and 20 mg Ketjen black were added to 100 mL of water, and the mixture was stirred, then heated to 100 °C. Next, 2.06 mL of 5 wt% trisodium citrate was added, then heated and stirred for 30 min; then, the solution was cooled to room temperature and stirred overnight. The resulting sample was separated by centrifuging, repeatedly washed with water and ethanol, and finally dried naturally overnight.

## 3. Results and Discussion

**Figure 1:**
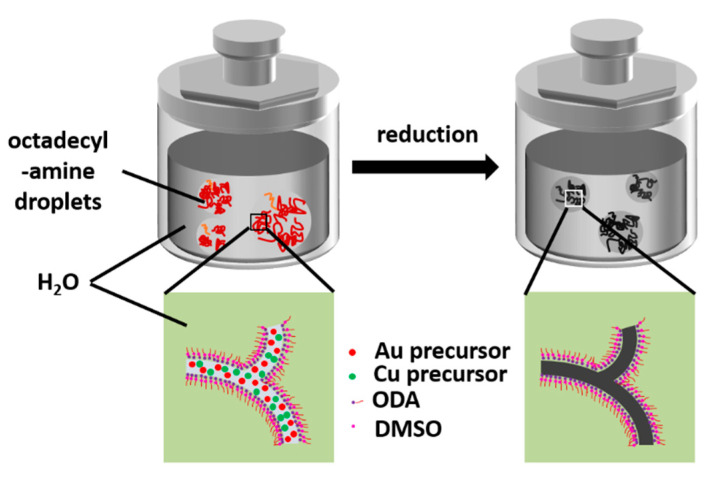
Schematic illustration of the preparation procedure of AuCu.

**Figure 2:**
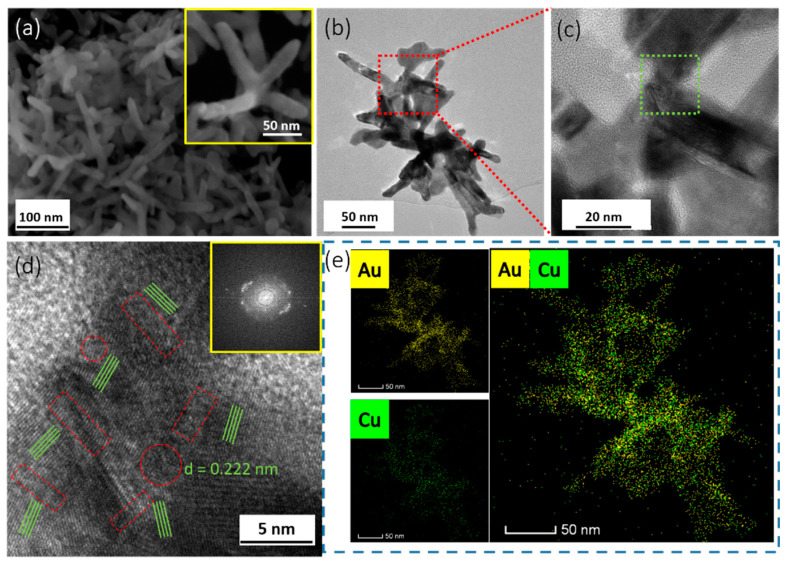
(**a**) SEM, (**b**,**c**) TEM, (**d**) enlarged HRTEM image of the green part in c, and (**e**) relative elemental mappings of AuCu.

**Figure 3:**
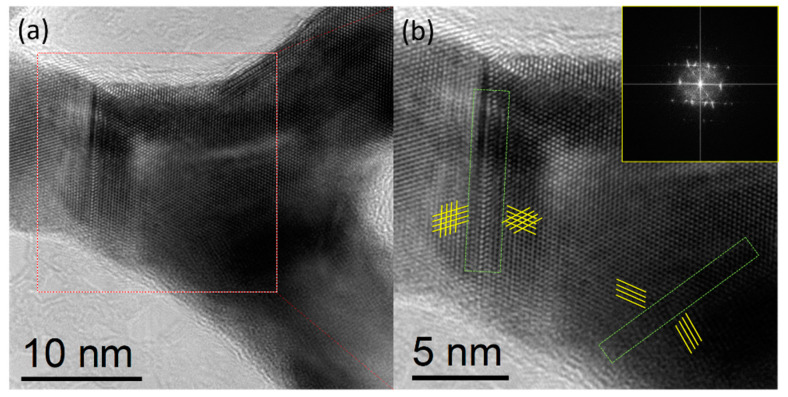
(**a**) HRTEM image of the kink in AuCu, and (**b**) relative enlarged HRTEM and FFT images of the red-white parts in (**a**).

**Figure 4:**
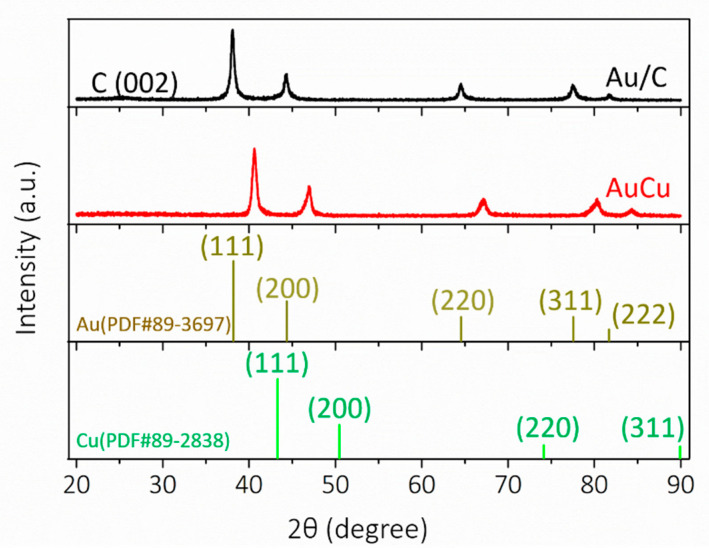
XRD patterns of AuCu and Au/C.

The process of AuCu synthesis through a W/O soft template method is shown schematically in Figure 1. Compared to conventional hard templates, soft templates are convenient to remove and have no need for surface modifications or functionalizations. Microemulsions, as a soft template, are therefore very convenient and efficient [22]. A microemulsion is usually a transparent or translucent liquid stable system composed of oil, water, surfactant, cosurfactant, and electrolyte. In detail, octadecylamine, with more lipophilic groups (−CH_3_) than hydrophilic groups (−NH_2_), was used as an oil phase and surfactant; DMSO as polar organic matter played the role of cosurfactant, and the precursors of Au and Cu as electrolytes were in water phase. In addition, octadecylamine was used as a reducing agent to reduce the precursor at 120 °C to form self-supported AuCu [23]. In this system, two immiscible continuous media were divided into small spaces by surfactant amphiphilic molecules to form a micro-reactor, the size of which can be controlled in the nanoscale, and the reactants reacted in the system to form solid substances. The microemulsion can control the size and stability of the nanomaterials accurately, and it limited the process of nucleation, growth, coalescence, and agglomeration [24]. The co-existence of Au and Cu precursors plays an important role in the formation of self-supported nanostructure. When only a Au precursor exists, only irregular large particles are formed, and in the case that only a Cu precursor exists, the sample cannot be synthesized (Figure S1). As shown in Figure 2a, the SEM images show that the self-supported AuCu presented a branched, porous nanostructure, which was further confirmed by the individual nanobranch, shown in the inset image. The TEM images in Figure 2b,c show the nanobranch consisting of several 13 nm skeletons, which connected in the kink of the structure. In more detail, the HRTEM, together with FFT images in Figure 2d, shows that different growth directions co-existed at the kink, and the twin boundaries and dislocations in Figure 2d and Figure 3 were observed; these abundant defects played an important role in electrocatalysis [25]. In addition, the interplanar distance was 0.222 nm (Figure 2d), and, according to Vegard’s law, the interplanar distance of an alloy is between the mono metals [26]. For AuCu, the interplanar distance is 0.222 nm, and for Au (111) and Cu (111), the theoretical interatomic distance of the (111) plane is 0.235 nm and 0.209 nm, respectively, and this is consistent with Vegard’s law, indicating the formation of an alloy phase [27]. The relative element distribution mapping in Figure 2e shows that Au and Cu were evenly distributed in the nanobranch, indicating the formation of a single-phase alloy. For Au/C, spherical gold nanoparticles were loaded on carbon (Figure S2). In Figure 4, the XRD pattern of Au/C shows a face-centered cubic lattice of Au with five peaks corresponding to (111), (200), (220), (311), and (222) planes, and the peak of C (002) was observed, confirming the formation of Au/C. Here, the peak of C (002) was weak, which was probably due to the relatively low temperature of the synthesis process, which contributed little to improving the degree of graphitization [28]. For AuCu, as the two metals form an alloy, their lattice will change, and the diffraction pattern will change accordingly. Here, the incorporation of Cu into the Au lattices shifted the peak positions to higher angles, indicating lattice contraction [27]. According to the Bragg equation [29], the interplanar distance of AuCu(111) is calculated to be 0.222 nm, and this is consistent with HRTEM analysis. In addition, there was no peak of a single element or relative oxide, which further verified the formation of a single-phase alloy.

**Figure 5:**
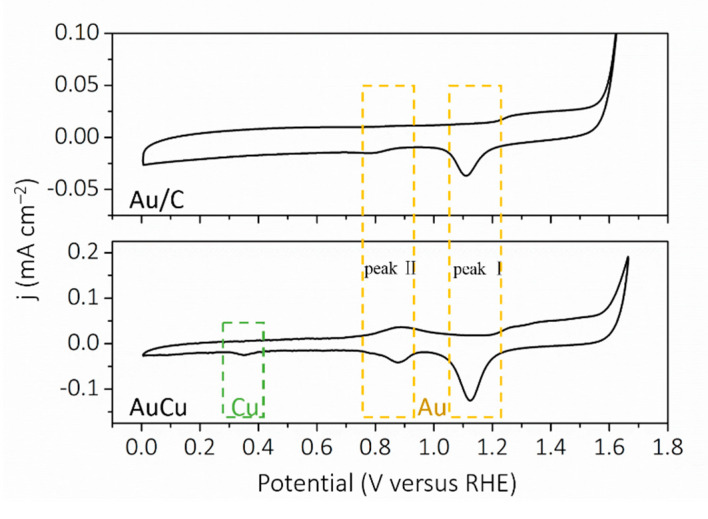
CV curves of AuCu and Au/C in Ar-saturated 0.1 M KOH at 10 mV s^–1^.

Due to the abundant defects in the self-supported AuCu materials, we explored the application of AuCu as an MOR and ORR electrocatalyst. As the surface state plays an important role in electrocatalysis, it was analyzed by the cyclic voltammetry (CV) method in Ar-saturated 0.1 M KOH at 10 mV s^–1^ [30]. As shown in Figure 5, for AuCu, reduction characteristic peaks of Au and Cu were observed on negative scanning; the peaks at 1.12 V and 0.88 V stand for the Au reduction characteristic peaks, and the peak at 0.35 V stands for the reduction characteristic peaks of Cu. For Au/C, the reduction characteristic peaks of Au were observed at 1.11 V and 0.78 V; and for the Au element, it is known that reduction peak Ⅰ at a higher potential represents the reduction of gold oxide, and reduction peak Ⅱ at a lower potential represents the desorption of OH^-^. The potential of peak Ⅱ of AuCu was obviously higher than that of Au/C, indicating that the intermediate species of Au in AuCu can be desorbed faster, which could enhance electrocatalytic activity [31].

**Figure 6:**
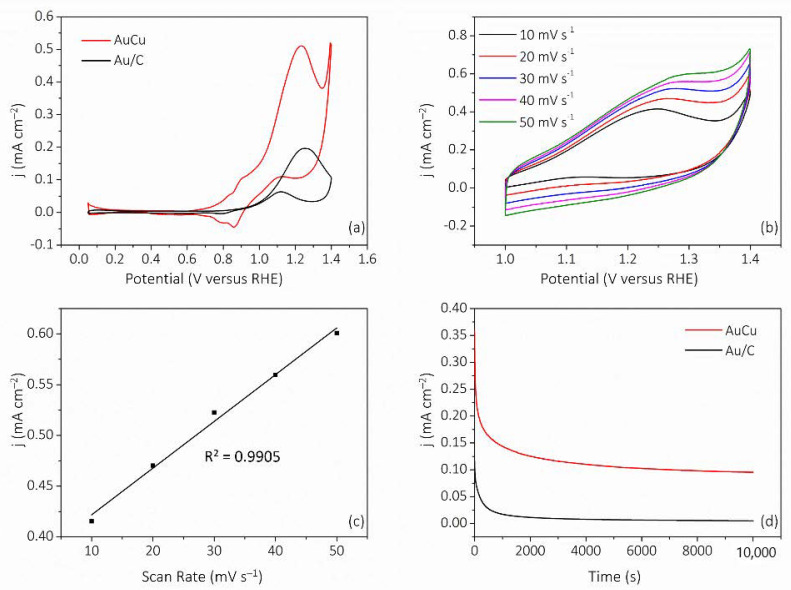
MOR electrocatalytic evaluation of AuCu. (**a**) CVs of AuCu and Au/C. (**b**) CVs of AuCu taken at various scan rates. (**c**) Calibration plot of the oxidation peak current density versus scan rate. (**d**) CA curves of AuCu and Au/C.

The MOR electrocatalytic activities of AuCu were evaluated against Au/C. Through a CV test with and without methanol (Figure S3), it was shown that both AuCu and Au/C have obvious MOR activity. Next, a background subtraction on the curves was performed in order to eliminate the interference of capacitance and surface metal redox current on methanol oxidation current. Figure 6a shows the CV curves taken in 0.5 M KOH with 2 M methanol saturated with Ar at a sweep rate of 20 mV s^–1^ after background subtraction, with obvious catalytic properties for MOR, including forward and backward oxidation peaks. The forward peak current of AuCu was 0.51 mA cm^–2^, 2.68 times greater than that of Au/C (j_Au/C_ = 0.19 mA cm^–2^). Additionally, the peak potential was 1.23 V, 20 mV smaller than Au/C (E_Au/C_ = 1.25 V). This demonstrates that AuCu possesses excellent MOR activity. Next, as shown in Figure 6b,c, we measured the forward oxidation peak current of AuCu at different scan rates, and obtained a good linear relationship between current density and scan rate, indicating that the electrocatalytic oxidation of methanol was governed by a surface-controlled process [32]. In addition, we tested the MOR stability of AuCu and Au/C via a 10,000 s- chronoamperometric (CA) test, and the results (Figure 6d) showed that the current density of AuCu was higher during the whole testing process. The above analysis showed that AuCu has excellent MOR performance.

**Figure 7:**
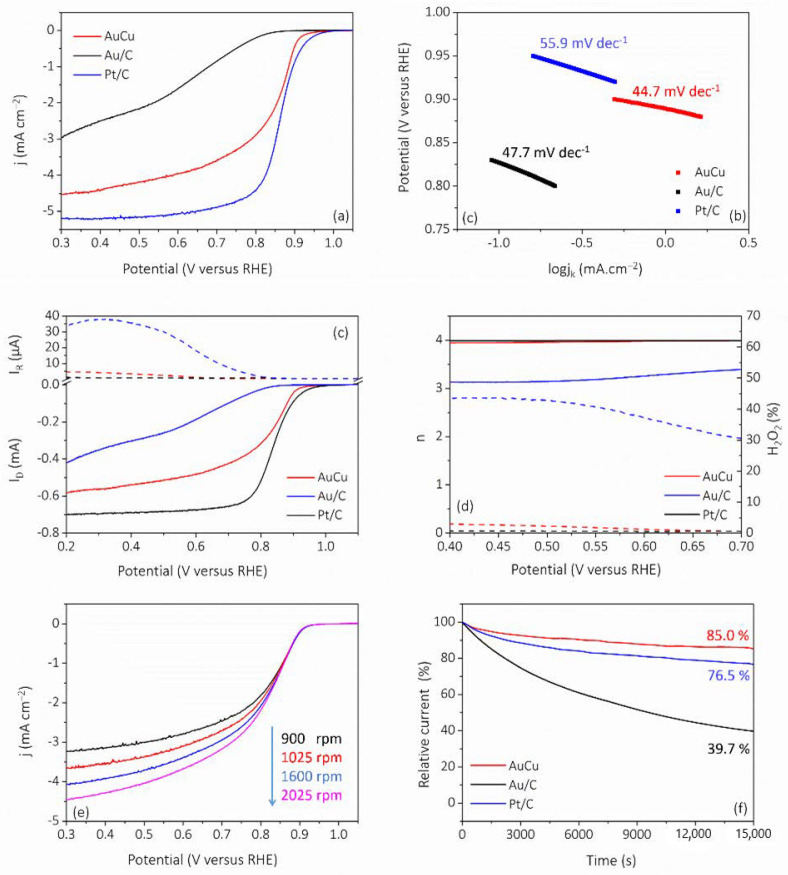
ORR electrocatalytic evaluation of AuCu. (**a**) LSV curves and (**b**) Tafel plots. (**c**) RRDE measurement at 1600 rpm and (**d**) corresponding n and H_2_O_2_% of AuCu, Au/C, and Pt/C. (**e**) LSVs of AuCu taken at various rpm. (**f**) CA curves of AuCu, Au/C, and Pt/C.

Then, the ORR performance of the catalyst was evaluated by linear sweep voltammetry (LSV). As shown in Figure 7a, the limiting diffusion current of AuCu was 4.5 mA cm^–2^, which was slightly lower than that of Pt/C (5.2 mA cm^–2^), while Au/C showed poor activity and did not possess a good limiting diffusion current platform. The half-wave potential of AuCu was 0.84 V, which was close to that of Pt/C (0.85 V). Next, the reaction kinetics of the catalysts were evaluated by Tafel slope. As shown in Figure 7b, the Tafel slope of AuCu was 44.7 mV dec^–1^, which was smaller than that of Au/C (47.7 mV dec^–1^) and Pt/C (55.9 mV dec^–1^), indicating that AuCu had faster reaction kinetics [33]. Then, the kinetic mass current was calculated as normalized by noble metal loading. As shown in Figure S4, it was found that the mass activity of AuCu (95.8 mA mg^–1^) was close to that of Pt/C (104.2 mA mg^–1^). In Figure 7c, the number of transferred electrons and the yield of intermediate products (H_2_O_2_%) were evaluated by the RRDE measurement, and Figure 7d shows that that the n and H_2_O_2_% of AuCu (n = 3.94–3.98, H_2_O_2_% = 2.94–0.57%) were close to those of Pt/C (n = 3.97–3.98, H_2_O_2_% = 0.65–0.63%), and significantly better than those of Au/C (n = 3.13–3.38, H_2_O_2_% = 43.4–30.5%), indicating the high ORR efficiency of AuCu. In addition, the ORR curves at different rotational speeds were obtained (Figure 7e). According to the Koutecky–Levich (K–L) curve, shown in Figure S5, the ORR transfer electron number of AuCu was 3.63–3.71, which was close to that of the four-electron process. Based on the results presented above, the relative characteristics and comparisons are summarized in Table S1. Next, we evaluated the stability of the catalyst by the CA method. As shown in Figure 7f, after the 15,000 s-CA test, the ORR activity of AuCu remained 85%, while Pt/C and Au/C showed a decrease of 23.5% and 60.3%, respectively, indicating the excellent stability of AuCu. The results illustrate that AuCu is a promising catalyst for ORR.

**Figure 8:**
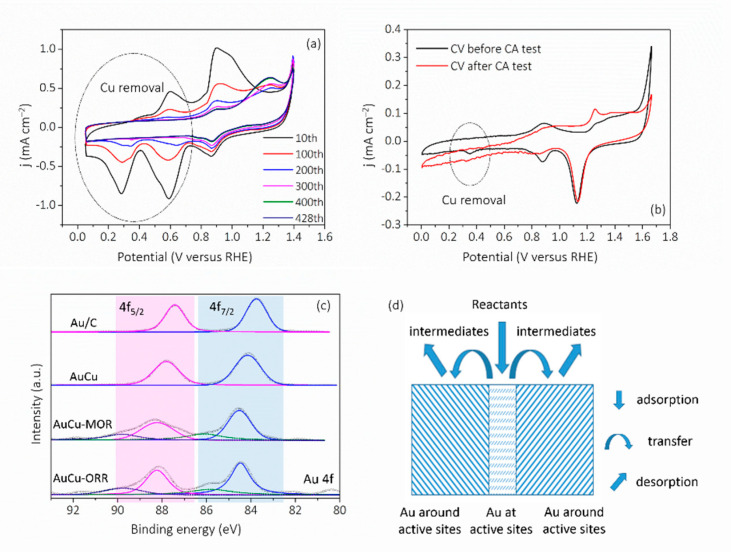
(**a**) CV curves of AuCu taken at various circles of MOR. (**b**) CVs of AuCu taken before and after ORR-CA. (**c**) High-resolution Au 4f XPS spectra. (**d**) Schematic diagram of reaction mechanism.

The reasons for the MOR and ORR catalytic performance of AuCu can be explained according to the following aspects. In terms of the overall structure of the catalyst, the self-supported structure is conducive to the transport of reactants and the improvement of the conductivity of the catalyst [34]. In more detail, a large number of defects in the kink are favorable for the adsorption of reactants; using MOR as an example, as shown in Figure 8a and Figure S6, in the electrochemical process, the removal of Cu from the surface occurred, which could lead to the formation of more low-coordinated Au and could enhance the adsorption of methanol on the active site and gradually improve the activity of MOR [35]. It is worth noting that in the AuCu system, Cu tends to dissolve in the presence of external force, which is very common in the phenomenon of dealloying [36]. In this study, with the progress of electrochemical reaction, Cu dissolution occurred, and the surface gradually changed from a AuCu alloy to a Au-rich surface, and Au became the main active site. In the above electrocatalytic part, the description of “AuCu catalyst” was used to keep consistent with the synthesis. In addition, the electronic structure of the active site also plays an important role in the catalytic activity, and XPS analysis was performed (Figure 8c and Figure S7). All samples showed two main peaks standing for Au^0^ (4f_7/2_) and Au^0^ (4f_5/2_), respectively [37]. Compared to Au/C (83.73 and 87.43 eV), Au in AuCu (84.10 and 88.80 eV) showed a positive shift, and after the removal of Cu (Figure S8) during the electrochemical process shown in Figure 8a,b, the Au in AuCu–MOR (84.52 and 88.23 eV) and AuCu–ORR (84.47 and 88.22 eV) showed a further positive shift, which indicates that after Cu removal, the adsorption ability of Au on the surface was weakened, in particular the Au neighboring the active sites in the kink (Figure 8d). According to previous theoretical DFT research, the lower metal–O affinity of the alloy would accelerate the desorption of the ORR products, implying some contribution to the activity enhancement for the ORR [17].

## 4. Conclusions

In this work, a self-supported defect-rich Au-based nanostructure was synthesized via a convenient method. As an electrocatalyst in an alkaline media, it showed efficient MOR and ORR performance, and the impacts of surface defects and electrochemical Cu removal on MOR and ORR performance were explored in depth. This study provides new insights to better understand and prepare Au-based electrocatalysts with high efficiency in the move towards alkaline methanol fuel cells.

## Data Availability

The data presented in this study are available on request from the corresponding author.

## Supplementary Materials

The following are available online at https://www.mdpi.com/article/10.3390/nano11092193/s1. Figure S1: (a) TEM image of the product when only a Au precursor exists; (b) optical photo when only a Cu precursor exists, which indicates the product cannot be synthesized efficiently (note: the white precipitate is octadecylamine); Figure S2: (a) TEM, (b) HRTEM, (c) HAADF-STEM image, and (d) relative elemental mappings of Au/C; Figure S3: (a) CV curves of AuCu with and without methanol, (b) CV curves of Au/C with and without methanol; Figure S4: Mass activities of Au/C, AuCu, and Pt/C; Figure S5: Corresponding K–L plots of LSVs of AuCu taken at various rpm; Figure S6: Anodic current density at 0.60V and 1.25V taken at various circles of MOR; Figure S7: ICP and EDS quantitative analysis of (a) Au/C and (b) AuCu; Figure S8: XPS spectra of Cu2p in AuCu-MOR, AuCu-ORR, and AuCu; Table S1: Summaries of the ORR catalytic characteristics of Au/C, AuCu, and Pt/C, respectively.
