# Supplementary material for: Self-Supported Defect-Rich Au-Based Nanostructures as Robust Bifunctional Catalysts for the Methanol Oxidation Reaction and Oxygen Reduction Reaction in an Alkaline Medium
Source: Nanomaterials (Basel). 2021 Aug 26;11(9):2193. doi: 10.3390/nano11092193 (PMC8467196; doi:10.3390/nano11092193)
Supplement: Supplementary file 1 [file nanomaterials-11-02193-s001.zip › nanomaterials-1325567-supplementary.pdf]

nanomaterials

MDPI

Supporting Information

Self-Supported Defect-Rich Au-Based Nanostructures as Robust Bifunctional Catalysts for the Methanol Oxidation Reaction and Oxygen Reduction Reaction in an Alkaline Medium

Yuanyuan Tao [1], Xiu Liang [1], Guanchen Xu [1], Dongwei Li [1], Yong Li [1], Na Zhang [2,*], Yingzhou Chen [3], Xifeng Jiang [4] and Hongyu Gong [1,*]

[1] Advanced Materials Institute, Qilu University of Technology (Shandong Academy of Sciences), Jinan 250014, China; yytao@sdas.org (Y.T.); xliang@sdas.org (X.L.); gcxu@sdas.org (G.X.); dwli@sdas.org (D.L.); yongli@sdas.org (Y.L.)
[2] School of Chemistry and Chemical Engineering, Shandong University, Jinan 250100, China
[3] Shandong Gold Smelting Co., Ltd., Laizhou 261441, Shandong, China; chenyingzhou1989@163.com
[4] Gweike Tech Co., Ltd., Jinan 250100, China; koki0201@163.com
* Correspondence: nzhang@sdu.edu.cn (N.Z.); hygong@sdas.org (H.G.)

Citation: Tao, Y.; Liang, X.; Xu, G.; Li, D.; Li, Y.; Zhang, N.; Chen, Y.; Jiang, X.; Gong, H. Self-Supported Defect-Rich Au-Based Nanostructures as Robust Bifunctional Catalysts for the Methanol Oxidation Reaction and Oxygen Reduction Reaction in an Alkaline Medium. *Nanomaterials* **2021**, *11*, 2193. https://doi.org/10.3390/nano11092193

Academic Editor(s):

Received: 19 July 2021
Accepted: 23 August 2021

Published: 26 August 2021

Publisher's Note: MDPI stays neutral with regard to jurisdictional claims in published maps and institu-tional affiliations.

Copyright: © 2021 by the authors. Submitted for possible open access publication under the terms and conditions of the Creative Commons Attribution (CC BY) license (http://creativecommons.org/licenses/by/4.0/).

Experimental

Chemicals

Nafion (5% w/w) and commercial Pt/C (20 wt %) were obtained from Alfa Aesar. Octadecylamine (95 wt %), $HAuCl_4.4H_2O$ (99 wt %), $CuCl_2.2H_2O$ (99 wt %), dimethyl sulfoxide (99 wt %), ethanol (99 wt %) and KOH (95 wt %) were obtained from Shanghai Aladdin Biochemical Technology Co., Ltd.

Material characterization

Scanning electron microscope (SEM) was performed on JSM-7610FPlus. Transmission electron microscope (TEM) was performed on FEI Talos F200s. X-ray diffraction (XRD) was characterized by Rigaku D/Max-r B. X-ray photoelectron spectroscopy (XPS) was performed on Thermo Fisher ESCALAB Xi+. Inductively coupled plasma mass spectrometer (ICP-MS) was obtained by PerkinElmer NEXION350X.

Electrochemical measurements

A CHI760E electrochemical workstation and RRDE-3A apparatus were employed to perform the electrochemical measurements at 25°C. For the electrode preparation, 1 mg AuCu was dispersed in a solution of 0.8 mL ethanol and 0.2 mL Nafion (0.2 % w/w in a mixture of ethanol), and the solution was ultrasonic mixed for 30 minutes. Next, for RDE (3 mm in diameter), 4 μL solution was dip-coated on the surface; for RRDE (4 mm in diameter), 6.8 μL solution was dip-coated on the surface. Au/C, Pt/C, served as the references, and precious metal loading of all working electrodes were identical based on ICP results (Fig. S7). After drying under ambient conditions, the RDE or RRDE was used as working electrode in the three-electrode system, an Ag/AgCl (saturated KCl solution) and a Pt foil were used as reference and counter electrodes, respectively. For MOR, the experiments were examined in a 0.5 M KOH + 2 M methanol, and for ORR, the experiments were examined in a 0.1 M KOH, and all potentials were referenced to the standard reversible hydrogen electrode (RHE) according to E (RHE) = E (Ag/AgCl, saturated KCl solution) + 0.198 + 0.059 pH. For LSVs, the linear sweep voltammetry (LSV) was performed from 1.1 to 0.2 V at 1600 rpm. For RRDE test, the ring potential was set at 1.2 V. For chronoamperometric (CA) test, the i-t curve was obtained under a constant potential of 1.25 V for 10,000 s for MOR, and 0.765 V for 15,000 s for ORR, respectively.

The RRDE test was applied to measure the hydrogen peroxide yield (% HO_2^-) and the electron transfer number (n) during ORR.

$$n = 4 \times \frac{abs(j_D)}{abs(j_D) + j_R / N} \tag{1}$$

$$\%HO_2^{\ -} = 100 \times \frac{2 j_R / N}{abs(j_D) + j_R / N} \tag{2}$$

where j_D and j_R could be determined from polarization curves directly, and the collection efficiency (N) was 0.424.

To get n, the rotating speeds were ranged from 900 to 2025 rpm and n was calculated according to Koutecky–Levich (K-L) equation (3) and (4):

$$1/j = 1/j_k + 1/j_d = 1/j_k + 1/(B\omega^{1/2}) \tag{3}$$

$$B = 0.62nFD_{O_2}^{\ 2/3}\upsilon^{-1/6}C_{O_2} \tag{4}$$

where j is the measured current density, j_k is the kinetic-limiting current density, j_d is the diffusion-limiting current densities, ω is the electrode rotation rate. D_{O_2} is the diffusion coefficient of O_2 (D_{O_2} = 1.86 ×10^{-5} cm^2 s^{-1}), F is the Faraday constant (96485 C mol^{-1}), C_{O_2} is the concentration of O_2 dissolved in electrolyte (C_{O_2} = 1.21 × 10^{-6} mol cm^{-3}), υ is the kinetic viscosity of the solution (υ = 0.01 cm^2 s^{-1}).

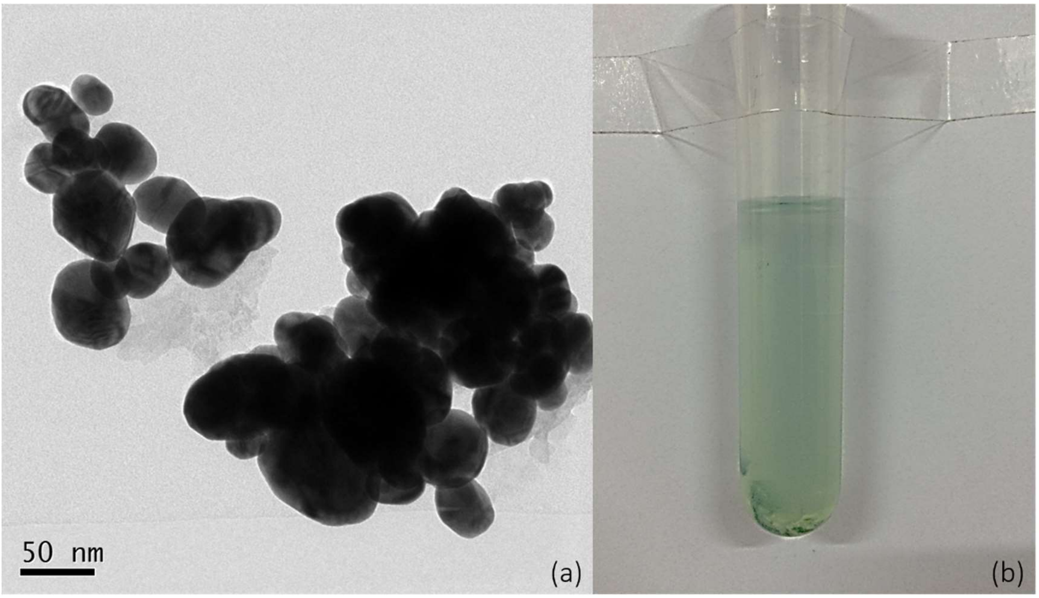

Figure S1. (**a**) TEM image of the product when only Au precursor exists, (**b**) optical photo when only Cu precursor exists, which indicate the product cannot be synthesized efficiently (note: the white precipitate is octadecylamine).

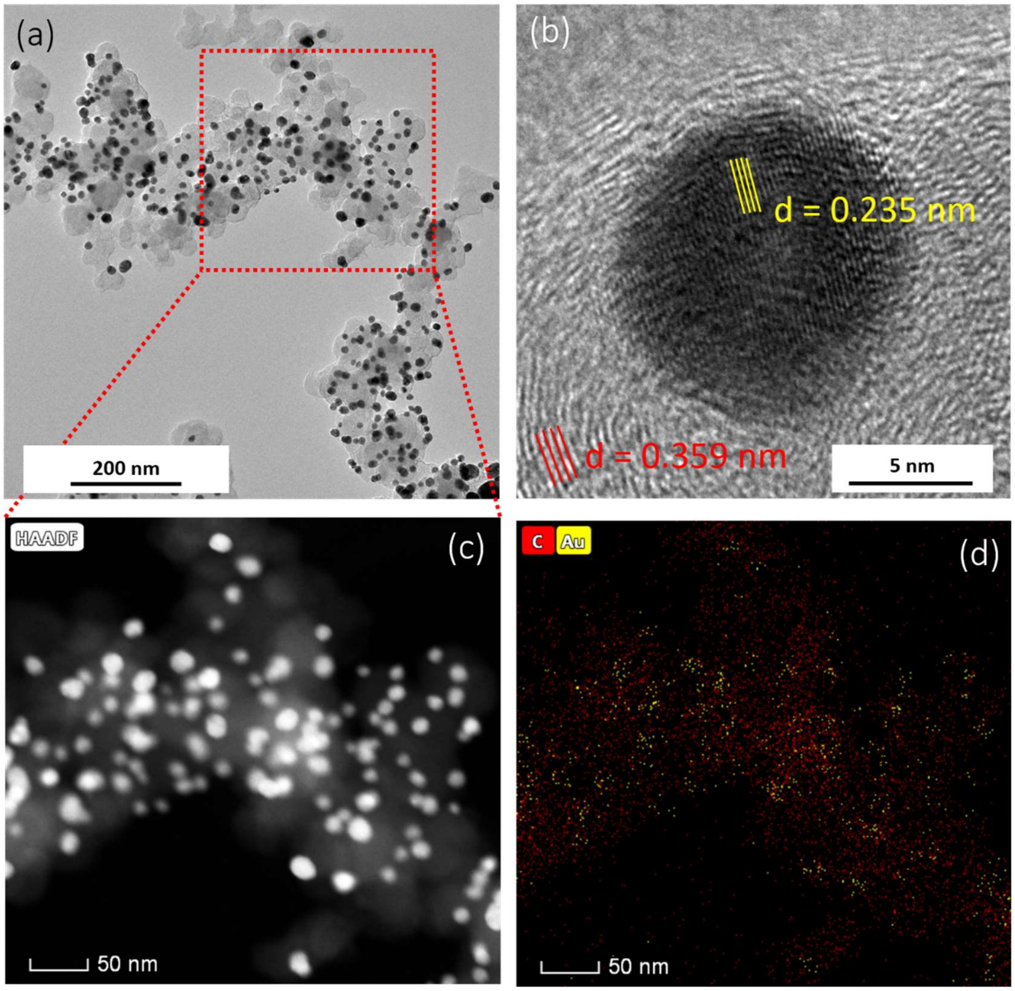

Figure S2. (**a**) TEM, (**b**) HRTEM, (**c**) HAADF-STEM image and (**d**) relative elemental mappings of Au/C.

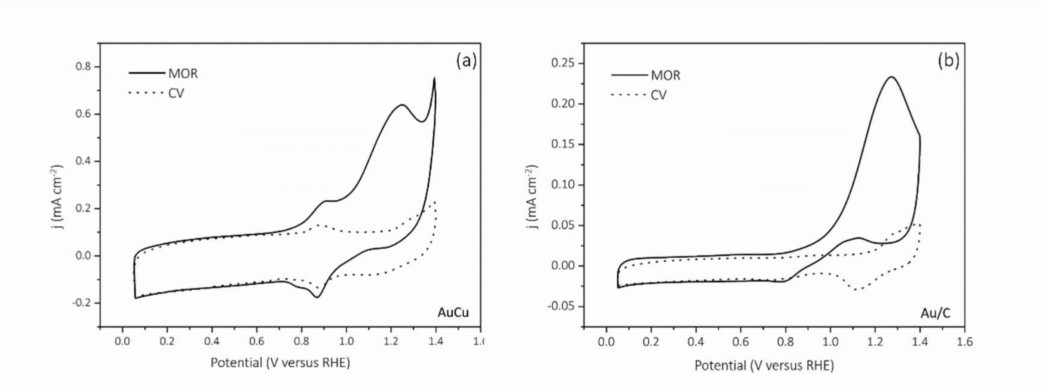

Figure S3. (**a**) CV curves of AuCu with and without methanol, (**b**) CV curves of Au/C with and without methanol.

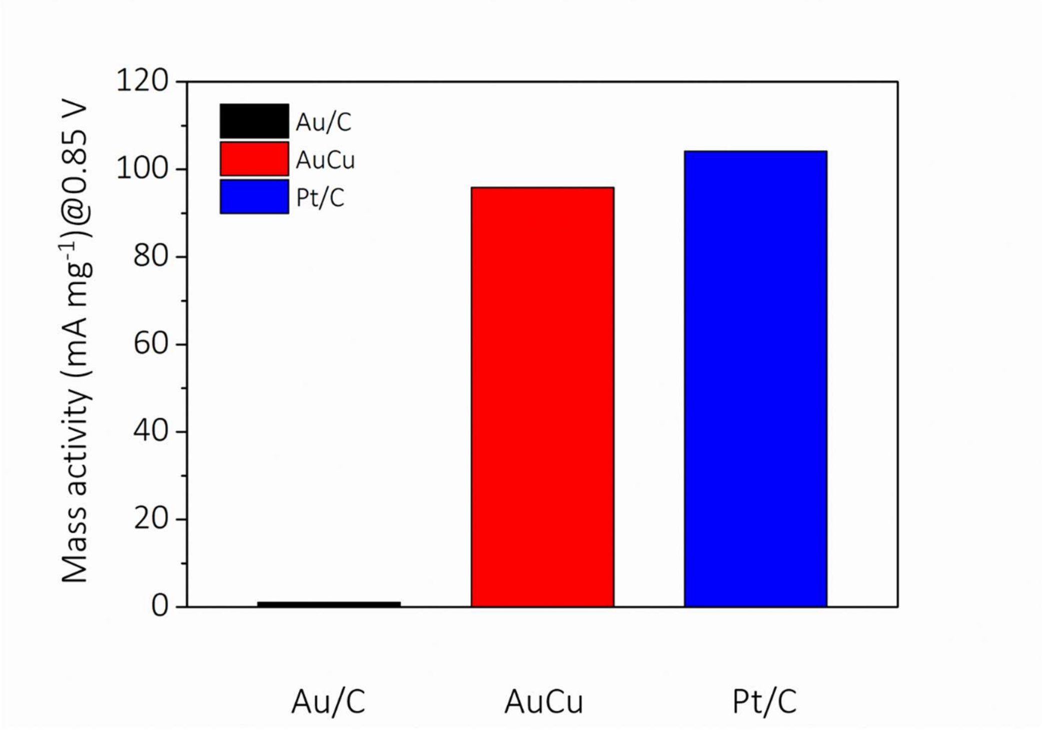

Figure S4. Mass activities of Au/C, AuCu and Pt/C.

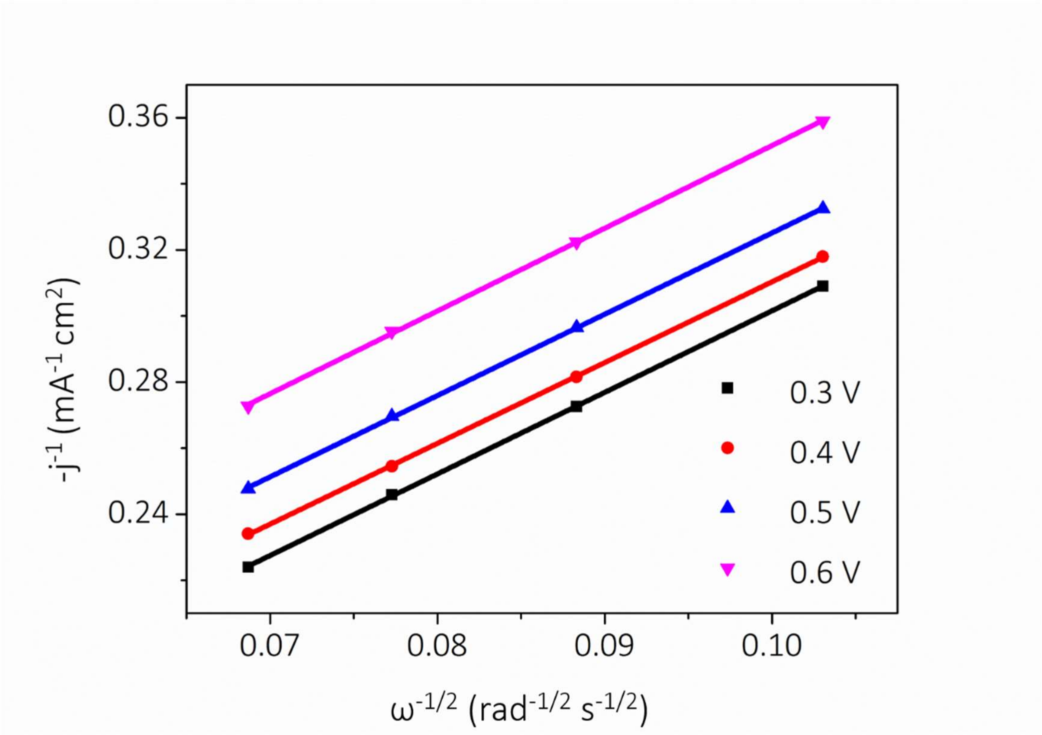

Figure S5. Corresponding K-L plots of LSVs of AuCu taken at various rpm.

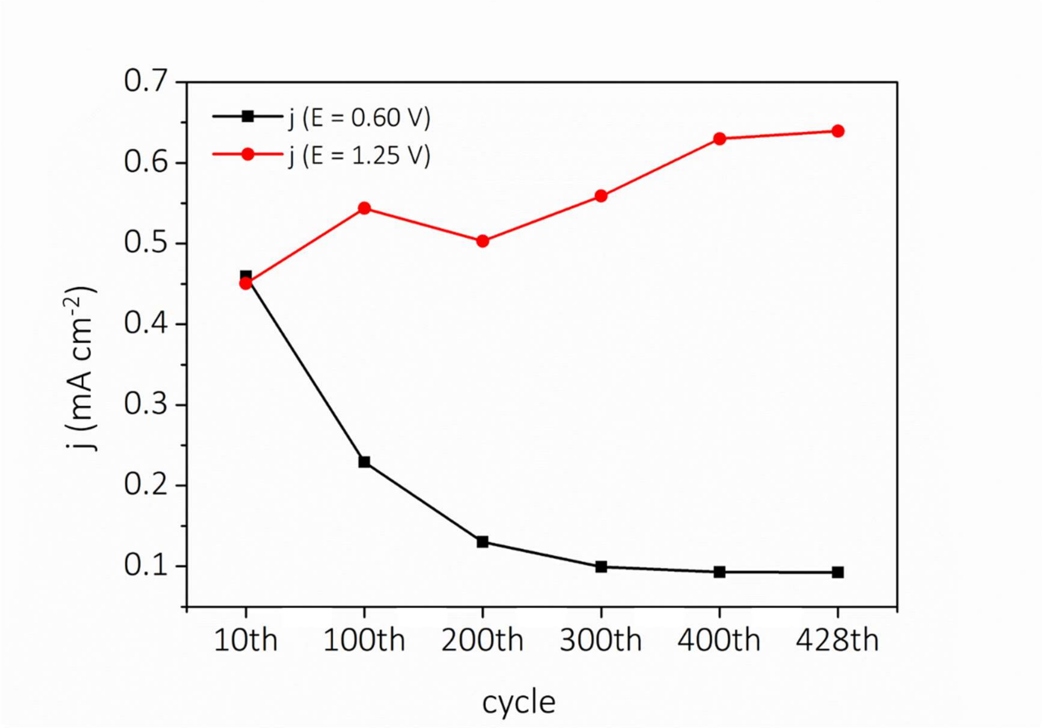

Figure S6. Anodic current density at 0.60V and 1.25V taken at various circles of MOR.

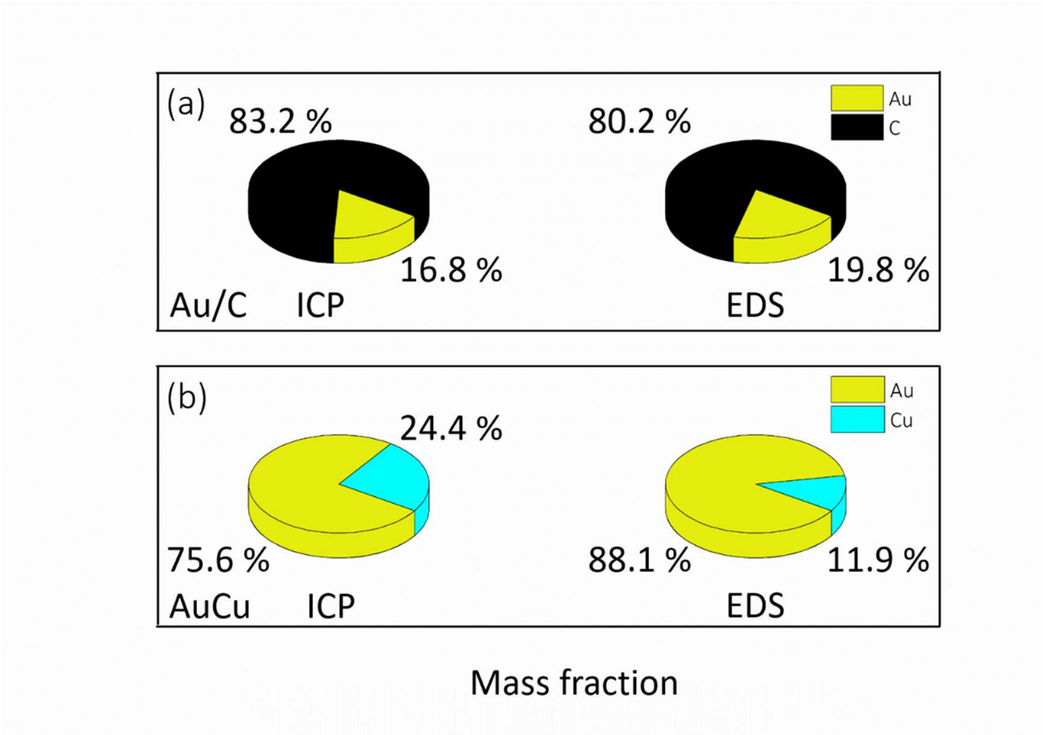

Figure S7. ICP and EDS quantitative analysis of (**a**) Au/C and (**b**) AuCu.

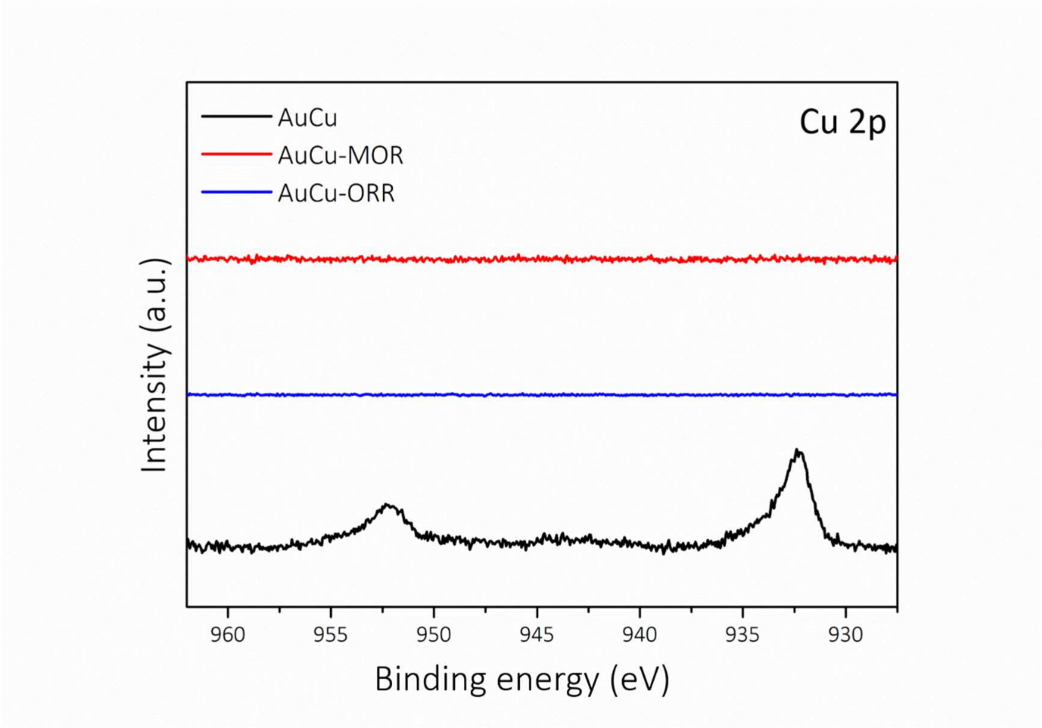

Figure S8. XPS spectra of Cu2p in AuCu-MOR, AuCu-ORR, and AuCu.

Table S1. Summaries of the ORR catalytic characteristics of Au/C, AuCu and Pt/C, respectively.

	**Onset potential for ORR (V)**	**$E_{1/2}$ (V)**	**Kinetic precious mass current at 0.85 V ($mA\ mg^{-1}$)**	**The number of transferred electrons**
Au/C	0.83	—	1.1	3.13-3.38
AuCu	0.94	0.84	95.8	3.94-3.98
Pt/C	0.96	0.85	104.2	3.97-3.98
